# The Pleiotropic Effect of Physical Exercise on Mitochondrial Dynamics in Aging Skeletal Muscle

**DOI:** 10.1155/2015/917085

**Published:** 2015-04-05

**Authors:** Elena Barbieri, Deborah Agostini, Emanuela Polidori, Lucia Potenza, Michele Guescini, Francesco Lucertini, Giosuè Annibalini, Laura Stocchi, Mauro De Santi, Vilberto Stocchi

**Affiliations:** ^1^Department of Biomolecular Sciences, Division of Exercise and Health Sciences, University of Urbino Carlo Bo, Via A. Saffi 2, 61029 Urbino, Italy; ^2^Department of Biomedicine and Prevention, University of Tor Vergata, Via Montpellier 1, 00133 Rome, Italy

## Abstract

Decline in human muscle mass and strength (sarcopenia) is one of the principal hallmarks of the aging process. Regular physical exercise and training programs are certain powerful stimuli to attenuate the physiological skeletal muscle alterations occurring during aging and contribute to promote health and well-being. Although the series of events that led to these muscle adaptations are poorly understood, the mechanisms that regulate these processes involve the “quality” of skeletal muscle mitochondria. Aerobic/endurance exercise helps to maintain and improve cardiovascular fitness and respiratory function, whereas strength/resistance-exercise programs increase muscle strength, power development, and function. Due to the different effect of both exercises in improving mitochondrial content and quality, in terms of biogenesis, dynamics, turnover, and genotype, combined physical activity programs should be individually prescribed to maximize the antiaging effects of exercise.

## 1. Introduction

Aging is associated with a generalized decline in all physiological functions, and between the ages of 30 and 70 we are likely to observe a 25–30% reduction in most functional capacities [1]. Unfortunately, this physiological condition become clinically relevant in more than 25% of people older than 85 years [2] and is referred to as “frailty.” This condition has been defined as a state of increased vulnerability to poor resolution of homoeostasis after a stressor event, which increases the risk of adverse outcomes [3–5]. In other words, an apparently small insult, such as a minor infection or surgery, results in disproportionate changes in the health state. Although either the brain or the endocrine and immune system can be affected by frailty, the aging skeletal muscle has been regarded as the key component of frailty (see Clegg et al. [6]). The physiological decline of skeletal muscle function with aging, referred to as “sarcopenia,” is characterized by a progressive loss of neuromuscular performance, skeletal muscle mass, and stem cell function associated with loss of strength. This intrinsic muscle weakness, also known as a deterioration in “muscle quality” has traditionally been attributed to impaired ATP production, decrease in fiber specific tension, reduced excitation-contraction coupling, and reduced neural drive [7].

Furthermore, it has been reported that adults over the age of 60 spend most of their waking hours, 8 to 12 hours per day, engaged in sedentary pursuits [8]. Inactivity accelerates muscle catabolism, mitochondrial dysfunction, and oxidative stress accumulation and reduces aerobic capacity [9]. These problems can lead to a “vicious circle” of muscle loss, injury, and inefficient repair, causing elderly people to become increasingly sedentary over time. Thus, it is imperative to implement preventive and therapeutic strategies to boost muscle mass and regeneration in the elderly and hence maintain and improve both their health and independence and prevent the occurrence of the frailty condition.

Current evidence certainly indicates that a regular exercise program reduces and/or prevents a number of functional declines associated with aging. Since, besides genetic, environmental, and nutritional factors, the lack of physical activity plays a major role in the pathophysiology of frailty [6], regular exercise has also the potential to reduce the incidence of this problematic expression of population aging. Older adults can adapt and respond to both endurance and strength training. Aerobic/endurance exercise helps to maintain and improve cardiovascular and respiratory function, whereas strength/resistance-exercise programs have been found to be helpful in improving muscle strength, power development, and function [10]. In this age group, a regular exercise program also reduces the risk factors associated with chronic disease, such cardiovascular disease, diabetes, and osteoporosis, improving overall health and helping to increase lifespan [11]. Together, these training adaptations greatly improve “muscle quality” and functional capacity on the elderly, thus improving their quality of life.

The present review aims to assess the role of exercise in enhancing mitochondrial function, biogenesis, dynamics, turnover, and quality control in aging muscle, as an area of research on bioenergetics and homeostasis, which has placed the mitochondria at the center of these processes. Exercise induces beneficial adaptations for metabolic homeostasis. This could lead to significant changes in lifestyle, which could slow down the progression of age-related muscle functional decline and could also allow us to identify molecular responses that may be useful as both therapeutic targets and for exercise prescription.

## 2. New Evidence Supporting the Mitochondrial Theory of Aging and the Role of mtDNA

Although several theories have been suggested to clarify the mechanisms mediating aging, the “Free Radical Theory,” proposed in 1956 by Harman, is by far the most popular. This theory proposes that aging depends on oxidative modifications caused by highly reactive compounds such as free radicals, the most important of which are reactive oxygen species (ROS) and reactive nitrogen species (RNS) [12]. Later this theory was revised, identifying the mitochondria both as the primary sources of ROS and the primary targets of ROS damage [13, 14]. This new hypothesis, also called the “Mitochondrial Free Radical Theory of Aging” (MFRTA), is mainly based on the accumulation of ROS-mediated mtDNA damage, which arises from a “vicious circle” of ROS production, mtDNA mutations, and mitochondrial dysfunction. However, this idea still leaves many unanswered questions.

López-Otín et al. describe nine tentative hallmarks for aging in different organisms, suggesting that the rate of aging is controlled, at least to some degree, by genetic pathways and biochemical processes conserved in evolution [15]. These hallmarks are genomic instability, telomere attrition, epigenetic alterations, loss of proteostasis, deregulated nutrient sensing, mitochondrial dysfunction, cellular senescence, stem cell exhaustion, and altered intercellular communication. Nevertheless, mitochondrial dysfunction remains a common denominator of aging and it is considered to be part of the compensatory or antagonistic responses to the damage caused by cellular aging.

More recently Shokolenko has carried out a critical analysis on the role of mtDNA in aging, providing evidence that goes against the existence of the “vicious circle” [16].

MtDNA mutations observed in aging are randomly distributed and vary in type according to whether they occur in mitotic and postmitotic tissues. Point mutations are mostly observed in mitotic tissues, while large-scale deletions occur in postmitotic tissues [17]. In aging cells the highest rate of point mutations is represented by transitions, with transversions and small deletions equally distributed. Nonsynonymous and frameshift/premature termination codon point mutations have a significantly elevated frequency, as well as the predicted pathogenicity, when compared with the same variants in general populations. Since there is not a selective advantage for deleterious point mutations more than 60% of cellular copies of a given mtDNA-encoded trait have to be affected by a pathogenic mutation in order to observe the phenotypic manifestation and the impairment of mitochondrial function. These levels indeed are not achieved in naturally aged tissues of animals or humans [18–21]; thus these relatively low mutation loads, though in a heteroplasmic state, cannot be the driving force of age related decline in mitochondrial function. Hence increased ROS production in aging leads to increased mtDNA mutagenesis, though at present we do not fully understand how oxidative mtDNA lesions are processed by mitochondria to produce mutations.

Moreover it has been suggested that oxidative stress stimulates the expression of endogenous mitochondrial DNA methyltransferases (DNMTs) responsible of epigenetic modifications able to regulate the mitochondrial transcription [22] and promotes the increase of 8-hydroxy-2′-deoxyguanosine (8-oxodG) both in the nuclear and in mtDNA genome [23, 24]. Similarly in nuclear genome the increase of 8-oxodG in CpG nucleotide [25] seems to diminish the ability of DNA methyltransferases (DNMTs) to methylate the adjacent cytosine [26]. In fact, it significantly reduces the affinity of the methyl-CpG binding domain proteins (MBDs), necessary for the 5mC-mediated transcriptional repression, to their recognition sequence, thus compromising the transcription. Nevertheless whether this mechanism is also operative in mitochondria and, if so, whether it leads to the same consequences observed in the nuclear DNA requires further investigation.

According to MFRTA ROS-induced mtDNA damage should increase ROS production and trigger the “vicious” circle. Instead most of mtDNA point mutations do not affect ROS levels, as observed in “mutator-mice” [27–30] and also in humans [31]. Mito-mice are a knock-in model of mice that age prematurely due to the accumulation of random mtDNA mutations. Based on this evidence Barja has recently proposed a new version of MFRTA that does not include the “vicious circle” [32].

It is of note that the most recent reports on mtDNA damage, ROS, and aging explain the increased susceptibility of mtDNA strand breaks (but not the oxidative base damage) induced by ROS as a mitochondria-specific mechanism that helps to preserve mtDNA integrity. Thus increased ROS production has now come to be viewed as an adaptive response of mitochondria [16], often called* mitohormesis*, to mitigate dangerous changes rather than representing an inevitable byproduct of mitochondrial respiration.* Mitohormesis* also increases stress resistance, maintains mtDNA levels, preserves mtDNA fidelity, enables cells to tolerate high levels of mtDNA mutations [33], and generally prolongs lifespan [34–36]. This represents a novel-upsetting paradigm, which explains the failure of antioxidants to delay aging in clinical trials [37, 38].

Also of particular interest is the recently described circulating mtDNA that seems to increase with age. Indeed, when released extracellularly, it can act as “damage-associated molecular pattern” agent and it has been recognized as a cause of inflammation that can significantly contribute to the maintenance of the low-grade, chronic inflammation observed in elderly people [39].

At present we need to accurately determine the signature of oxidative stress in mitochondria; thus the development of reliable methods to detect the identity and the rate of ROS generated* in vivo* will allow us to gain further insights into the contribution of mtDNA in aging.

## 3. Aging Skeletal Muscle

Human aging is associated with both loss of muscle mass and structural alteration of the neuromuscular components resulting in impaired contractile function. Sarcopenia refers to the loss of muscle mass and, consequently, strength, with aging. The sarcopenic phenotype is characterized by a reduction of muscle mass, a shift in fiber-type distribution, associated with the loss of the ability to generate force, and hence an inability to effectively perform the activities of daily living (ADL). The phenotype does not necessarily include malaise. It has recently been confirmed that differences in the leg muscle cross sectional area (CSA) between young and elderly men can be attributed to differences in type II muscle fiber size [40]. In addition, it has been shown that aging is associated with the replacement of muscle fibers with intramuscular fat and connective tissue and with oxidative stress, degeneration of the neuromuscular junction, and changes in muscle metabolism, which lead to progressive loss of muscle function and frailty. Concomitant with shift in fibre-type distribution, a shift in energy metabolism seems to anticipate the onset of sarcopenia: indeed, activity of cytochrome c oxidase that it is involved in mitochondrial oxidative phosphorylation significantly decrease and the subcellular organization of mitochondria in oxidative fibre results compromised [41] (see paragraph “*Aged-related changes in skeletal muscle mitochondria*”).

Changes in the endocrine muscle microenvironment during aging might contribute to the progression and reduced reversibility of sarcopenia. Thus, for example, during aging production of insulin-like growth factor (IGF-I) and other anabolic molecules such as cytokines in muscle tissue declines, which could explain the reduced synthesis of myofibrillar and mitochondrial proteins with age [42]. It is known that muscle-specific IGF-I overexpression and its receptors attenuates the age-related loss of muscle mass [43] and has long been recognized as one of the critical factors for coordinating not only muscle growth, but also enhancing muscle repair and increasing muscle mass and strength [44].

IGF-I has also been found to contribute to oxidative balance and to mediate protective responses against oxidative stress* in vivo* [45]. IGF-I is a peptide hormone with a complex posttranscriptional regulation and generates distinct isoforms, namely, IGF-IEa, IGF-IEb, and IGF-IEc [46]. In murine models, the local muscle isoform of IGF-I (mIGF-I, the orthologue of human Mechano Growth Factor) has been shown not only to activate proliferation of myoblasts [46], but also to protect cardiomyocytes from oxidative stress via the Sirtuin 1 deacetylase activity [47]. However, chronic IGF-I supplementation might not be a healthy treatment for sarcopenia and cachexia since high level of insulin-IGF-I signaling, at least circulating, has been shown to favor cancer growth and to shorten lifespan [48].

In general, aged muscles are less responsive to anabolic and catabolic stimuli than young muscles. Indeed, menopause typically induces the reduction of about 15% of muscle mass and a decrease in estrogens levels in women, thus playing a potential role in this decline. Sarcopenia is known to be the result of unfavourable and detrimental effects, which involve hormonal, biological, nutritional, and physical activity-related mechanisms. It is challenging to determine the relative contribution of sex hormones on the sarcopenia establishment because the effect of hormonal supplementation to treat or prevent sarcopenia seems to be contradictory. On the contrary, it remains evident that the decline in muscle mass is related to an increased risk of functional impairment and physical disability [49]. Despite the rapid drop down of estrogens in women with menopause, serum testosterone levels in men decline lightly progressive starting from the third decade. Testosterone increases muscle protein synthesis and its effects on muscle are modulated by several factors, including genetic background, nutrition, and exercise. A recent review showed that testosterone supplementation produced an inferior increase of muscle CSA than resistance training alone [50]. Glucocorticoids have a reduced catabolic effect in aged sarcopenic rats and do not succeed in enhancing protein breakdown [51]. The cellular mechanisms at the base of altered sensitivity to anabolic and catabolic stimuli in aging and the physiological consequences of such reduced sensitivity to extrinsic factors remain thus unclear.

Data from the Baltimore Longitudinal Study of Aging [52] and the Health ABC study [53] showed that during aging muscle strength declines faster than muscle mass, suggesting a decrease in muscle “quality.” A decrease in type II muscle fibers is specifically related to overall muscle “quality.” In fact, evidence indicates that, as we age, peak force significantly decreases in these fibers and not in type I fibers [54]. Likewise, the increase in muscle mass that occurs following prolonged resistance-type exercise training (RT) can be entirely and unambiguously attributed to type II muscle fiber hypertrophy [55]. Therefore, any intervention counteracting sarcopenia should target type II muscle fiber hypertrophy by means of RT. Exercise programs, which include daily RT, should aim to prevent older, high-risk people from falling and to improve and maintain their functional capacity [56].

Both maximal rate of oxygen consumption (VO_2_) and resting VO_2_, when corrected for lean mass [57], decline with age in healthy individuals. Furthermore, since sedentary older people have a low arterial-venous difference, that is, their muscle oxygen extraction is reduced compared to younger adults at the maximal VO_2_ rate, it follows that both muscle mitochondrial content and function are reduced in the elderly [9]. A sedentary lifestyle, typical of the elderly, accelerates skeletal muscle dysfunction, which is not only due to simple reduction in muscle mass. Intrinsic muscle weakness, also known as deterioration in “muscle quality,” has traditionally been attributed to a decrease in the specific tension of the fiber and reduced neural drive. Calcium (Ca^2+^) accumulation in energized skeletal muscle mitochondria has emerged as a biological process of great physiological relevance [58]. The reduced amount of Ca^2+^ ions available to tolerate muscle contractions and impaired ATP production due to increased muscle utilization [59] are crucial to explaining the reduced strength and resistance to fatigue of skeletal muscle in response to exercise that occurs during aging. The malfunction of excitation-contraction coupling, the mechanism linking the action potential to Ca^2+^ release from the sarcoplasmic reticulum, is probably caused by the age-related decrease in the number of calcium release units (CRUs) as described by Zampieri et al. [60]. Moreover, low ATP generation could depend on mitochondrial impairment and misplacement in the microdomain of the CRUs crucial to mitochondrial membrane potential signal activation and for efficient ATP production [61] (see paragraph “*Aged-related changes in skeletal muscle mitochondria*”). Given that, the molecular identity of mitochondrial Ca^2+^ uniporter (MCU) has been discovered only recently [62]; the identity of the specific complex of anchoring that stabilizes the Mitochondrial-Sarcoplasmic Reticulum is still unknown. Increased intramyocellular lipid (IMCL) content and interfiber fat infiltration is associated with aging and inactivity. Older adults have larger IMCL droplets, higher content in the subsarcolemmal area, fewer mitochondria, and a lower proportion of IMCL in contact with mitochondria compared with IMCL occurring in young skeletal muscle. These factors likely contribute to age-related reductions in mitochondrial function and oxidative metabolism [63]. Magnetic resonance spectroscopy studies have shown that IMCL levels vary with insulin sensitivity and obesity, which is a common clinical picture in the older adult, and that inactivity combined with overconsumption of fat can have detrimental effects on muscular insulin sensitivity [64]. Endurance training (ET) increases mitochondrial content/activity and IMCL content in young, active men and women. ET induces positive changes in mitochondrial function and lipid oxidation and induces intracellular IMCL reorganization, which is reflective of a greater IMCL turnover capacity in both lean and obese women [65].

In accordance, the enzyme phosphoenolpyruvate carboxykinase (PEPCK), mainly linked to gluconeogenesis, has been recently associated with a prolonged lifespan. Eukaryotes have a gene for both a mitochondrial (PEPCK-M) and cytosolic (PEPCK-C) form of the enzyme. Skeletal muscle has a small but significant level of PEPCK-C activity [66]. There have been several proposals regarding the metabolic role of this enzyme in muscle, among these we have the production of pyruvate for the synthesis of alanine by alanine aminotransferase; another possible metabolic role of PEPCK-C in skeletal muscle is glyceroneogenesis. In order to determine the metabolic role of PEPCK-C in skeletal muscle, transgenic mice were generated (PEPCK-C^mus^ mice) [67]. During exercise, the PEPCK-C^mus^ mice mainly use fatty acid as the primary fuel, the stimulation of the aerobic metabolism in the PEPCK-C^mus^ mice could be due to the increased number of mitochondria noted in their skeletal muscle [67]. Another interesting feature of the PEPCK-C^mus^ mice is that they lived almost two years longer than the controls and had normal litters of pups at 30 to 35 months of age [67]. This evidence is very interesting because these mice violate the idea that limiting food intake increases longevity, as a matter of fact, the PEPCK-C^mus^ mice eat almost twice as much as controls. Altogether, these data suggest that sustained activity could be a key element to extend lifespan and counteract sarcopenia.

*In this contest, is sarcopenia reversible?* Several pharmacological treatments, including selective androgen receptor molecules, anti-myostatin antibodies and activation of notch-mediated satellite cell proliferation, have been recently explored [7]. Individual physical activity history and aerobic and resistance training interventions, which are the major focus of this review, seem to be associated with the reversibility of sarcopenia. Indeed, recent findings suggest that regular skeletal muscle contraction, such as a resistance training program of at least 12 weeks [68] or a combination of ET and RT activities [69] counteract the detrimental effects of a sedentary lifestyle, as well as the regular use of neuromuscular electrical stimulator [70]. Indeed, they represent a good intervention to attenuate and slightly reverse the decline of skeletal myofiber size, strength, and power associated with the ultrastructural disorders observed during aging. In agreement with those evidences, Melov et al. [71] highlighted that six months of resistance exercise training markedly reversed the transcriptional profile of elderly back to that of adulthood; this response was observed for most genes that are affected by both age and exercise, with a general improvement for transcripts related to mitochondrial function. Moreover a recent 4-year follow-up study on Chinese elderly people confirmed that the accomplishment of sarcopenia reversibility is associated with several lifestyle-related factors. In particular a high BMI resulted protective against sarcopenia occurrence; however, the increment of physical activity and the maintenance of a healthy weight was beneficial in the prevention of sarcopenia as well [72]. Further studies in different populations and with a longer follow-up are necessary to better investigate to what extent lifestyle behaviours might contribute to sarcopenia reversibility.

## 4. Aged-Related Changes in Skeletal Muscle Mitochondria

In the elderly, a significant proportion of the skeletal muscle mitochondria alter their ultrastructure and subcellular localization. Indeed, in older people, mitochondria in skeletal muscle appear enlarged, more rounded in shape, with matrix vacuolization and shorter cristae [73] by comparison with mitochondria found in young people. Moreover, a greater proportion of mitochondria in the elderly are depolarized or nonfunctional, which may be indicative of defects in mitochondrial turnover [74]. The density of mitochondria in skeletal muscle also drops considerably with aging [75], as shown, for example, by means of electron microscopy in the* vastus lateralis* muscle of people over 60 years of age when compared to their younger counterparts [60, 76].

Boncompagni et al., with an elegant study, observed a decrease in the frequency of CRUs at sarcomere's I-A band transition and tethered mitochondria [61] with aging, as mitochondria are reduced in terms of content, function, and turnover in both subsarcolemmal and intermyofibrillar pools [77]. In addition, muscle aging is associated with the progress of a segregated SR Ca^2+^ pool that uncouples from the E–C coupling machinery. The dynamic nature of Ca^2+^ sparks appears to be misplaced in aged skeletal muscle. This condition may lead to excessive mitochondrial ROS production and alteration of the cellular redox state that contributes to change Ca^2+^ release/reuptake [78, 79]. Changes in the E–C coupling apparatus and [Ca^2+^]i homeostasis may act as causal factors of, or adaptive responses to, muscle aging. Since mitochondria-CRUs cross talk seems to be crucial for efficient ATP production, impairment in ATP production may depend on mitochondrial dysfunction and possibly reduced number and misplacement. Ultrastructural data showed in the article by Zampieri et al. [70] indicate that CRU is better preserved in subjects who exercise regularly than in sedentary individuals. Mitochondrial occurrence appears higher in athletic than in sedentary seniors with parameters similar to those of healthy young subjects. Indeed a dual amelioration, that is, a mitochondrial higher frequency and an improved positioning of both organelles, is observed. Thus lifelong physical activity may counteract age-related decline of muscle functional output and muscle fibre ultrastructure. The decrease in skeletal muscle mitochondria is associated with an overall decline in mitochondrial dynamics and impairment of AMP-activated protein kinase (AMPK), which stimulates biogenesis and functionality [80]. However, the causes of this impairment are poorly understood.

How mtDNA damage, previously described as generally linked to ROS accumulation (see paragraph “*New evidence supporting the mitochondrial theory of aging and the role of mtDNA*”), may affect mitochondrial function is still open to debate both in the context of general cellular homeostasis and in sarcopenia [73]. Is mtDNA damage a consequence or a cause of the muscle deterioration process associated with aging? On one hand, mitochondrial energy reduction occurring before mtDNA mutations is often detectable. On the other hand, several studies in humans reveal strong correlations between mtDNA mutation rates and bioenergetic deficiency (typically complex IV) or muscle fiber atrophy [73].

Moreover, recent studies on aging skeletal muscle report that complex I and complex IV activities decrease substantially, probably because these two complexes have more of their subunits encoded by the more vulnerable mtDNA than the other complexes [73]. Age-associated mitochondrial dysfunction leads an accumulation of ROS and oxidative modification to macromolecules including proteins and failure of protein maintenance and turnover. As previously described, complexes I and III are the mitochondrial electron transport chain (ETC) sites of major ROS production. It was hypothesized that proteins of the ETC complexes are primary targets of ROS-mediated modification damaging structure and function and decline in tissue function [81]. An increased oxidative damage at the total proteome level is supposed to have a causative role in cellular aging [82], even if oxidatively modified proteins (Oxi-Proteome) have not been completely identified. In this regard, Baraibar et al. [83] have recently generated a database of proteins, which have been identified as increasingly carbonylated or modified by the lipid peroxidation product 4-hydroxy-2-nonenal (HNE) and by glycation (AGE) during aging, showing a conservation of several targeted molecules in different tissues and organ systems. Ahmed et al. [84] identified modified proteins using a proteomic approach coupled with immune-detection of HNE-, AGE-modified, and carbonylated proteins during replicative senescence of WI-38 fibroblasts. They identified by mass spectrometry thirty-seven proteins involved in quality control, energy metabolism, and cytoskeleton, showing that almost half of them were found to be mitochondria-related, underlining the susceptibility of mitochondria to senescence. A better knowledge of the Oxi-Proteome will allow to fully understanding the biological significance of these modifications [85]. However, it has become increasingly clear that most of the declines in mitochondrial biogenesis, turnover, and function are a consequence of physical inactivity. Indeed, when physical activity levels are matched between young and elderly people, or physical activity is otherwise taken into account, most investigations do not find any age-related changes in mitochondrial enzyme activities, mitochondrial respiration, or ATP flux [75, 86, 87].

To date, the endocrine mediators involved in mitochondrial (dys)function in skeletal muscle have not been deeply investigated. Recently, Puche et al. [88] demonstrated that IGF-I replacement therapy, in aging, induced mitochondrial protection, suggesting an IGF-I mediated cytoprotection effect (verified at least in neurons and hepatocytes). The authors demonstrated that the administration of low doses of IGF-I in elderly people, in which circulating IGF-I serum levels result as low as 50% compared to healthy older adults, was able to exert many beneficial effects on age related-changes, such us increasing testosterone levels, improving insulin resistance and lipid metabolism, and reducing oxidative damage on brain and liver. These benefits seem to be associated to mitochondrial protection mechanisms induced by the restore of IGF-I circulating levels. In agreement with this evidence, also Hernández-Aguilera and collaborators [89] showed that mitochondrial defects are linked to the IGF-I mTOR pathway, which is essential for the regulation of numerous processes, including cell cycle, energy metabolism, immune response, and autophagy.

Moreover, the study of steroid mediators of mitochondrial (dys)function in skeletal muscle is an emerging field and only few recent papers give some insight. For example, current studies demonstrate that mitochondria are important for the initial step of steroidogenesis, and sex steroid hormones are able to modulate mitochondrial biogenesis and function [90]. Indeed, the detection in mitochondria of glucocorticoid, steroid, and thyroid hormone receptors suggests their potential direct role in mitochondrial functional regulation [91, 92]. Dysregulation of mitochondrial function and sex steroid hormone action may compromise the maintaining cellular physiology and integrity and lead to a progressive decline in tissue function, accelerating the aging-associated phenotypes [90]. Reduction in specific endocrine regulation and accumulation of mitochondrial damage may create a feedback loop that favours the degeneration of tissue function during aging. Therefore, muscle cell changes associated with endocrine alterations and mitochondrial dysfunction require further attention.

## 5. Decreased Mitochondrial Dynamics and Quality Control Events

Mitochondria are highly dynamic organelles organized in a tubule reticulum. They constantly exchange components during biogenesis, fusion-fission events [93] which can be drastically changed by aging. In particular, the mitochondrial biogenesis signaling activated by the peroxisome proliferator-activated receptor gamma coactivator (PGC-1) family of cotranscription factors is reduced with increasing age [73]. The overexpression of PGC-1*α* in the skeletal muscle of aged mice improves oxidative capacity, suppresses mitochondrial degradation, and prevents muscle atrophy [94].

Building on our group's previous research in this area [95, 96], we have recently studied the role of PGC-1*α* in C2C12 myoblasts subjected to oxidative stress during the early stages of differentiation, as well as the effect of H_2_O_2_ (0.3 mM) on PGC-1*α* expression and its relationship with AMPK activation. We found that 1 h treatment with H_2_O_2_ causes mitochondrial fragmentation and a mild mitochondrial impairment with a consequent marked increase in PGC-1*α* mRNA expression, as described by Kang et al. [97] and Irrcher et al. [98]. In this condition, we also found an increased phosphorylation of AMPK compared to untreated cells. This suggests that oxidative stress may induce PGC-1*α* through the AMPK signaling pathway, probably in order to activate a defense-oriented signaling cascade (unpublished data).

However, although the defense signaling activation by AMPK phosphorylation was challenged, C2C12 myoblasts rapidly displayed a 30–40% reduction in their viability as well as a survivors' reduced differentiative efficiency during the postchallenge incubation stage (up to 7 days of culture). This observation implies that, in addition to probably being an obligatory and physiological response to ROS, activation of AMPK and of PGC-1*α* may not be sufficient to afford complete protection to cells against overwhelming oxidative stress. Thus increased mitochondrial production of ROS is involved at multiple levels in promoting apoptosis in skeletal muscle cells, an event which is part of the etiology and progression of numerous pathologies including sarcopenia and muscle disuse atrophy, as well as aging.

Romanello et al. [99] provided direct evidence of the importance of the existence of mitochondrial fragmentation as an amplifying circle in muscle atrophy. The mitochondrial network fragmentation induces energy, imbalance, which activates a FoxO3-dependent atrophy program through the AMPK pathway. Thus, mitochondria play a crucial role in catabolic muscle signaling: the mitochondrial fragmentation activates the AMPK-FoxO3 axis, which induces the expression of atrophy-related genes, protein breakdown, and muscle loss. The dual role of AMPK has been reviewed by Mihaylova and Shaw [100]. Authors described that activation of AMPK promotes the mitochondrial biogenesis via PGC-1*α* upregulation and simultaneously triggers the destruction of existing defective mitochondria through mitophagy. However, under energy stress conditions, the ROS-positive feedback loops on FoxO3 activity is acutely enhanced by AMPK sustaining autophagy and protein breakdown, both strongly related to muscle atrophy during myopathies and sarcopenia [99].

Mitochondrial fusion and fission contribute to mitochondrial function by exchanging components such as membrane, proteins, and DNA. Mitochondrial fission and fusion are regulated by GTPases of the Dynamin family, with opposite functions. Fission is mediated by dynamin related protein 1 (Drp1) and plays a key role in maintaining mitochondrial quality and mtDNA integrity, as it allows dysfunctional mitochondria to be severed from the network and to be removed by autophagy. Fusion is controlled by optic atrophy 1 (Opa1), mitofusin 1 (Mfn1), and mitofusin 2 (Mfn2 [101, 102]). Opa1 is also involved in degradative processes; indeed it regulates apoptosis by keeping the inner mitochondrial cristae junctions tight to prevent cytochrome c release, which characterizes apoptosis [103]. Few recent studies have reported that Mfn2 gene expression is lower in the skeletal muscle of older humans [63].

Of particular interest are data from muscle-specific Mfn1- and Mfn2-knockout mice. In these mice, researchers noted an enhanced mitochondrial proliferation and increased mutations in depletion of mtDNA and these changes occurred with accelerated muscle loss [33]. Age-associated changes in the dynamic remodeling processes of fission and fusion likely affect mtDNA integrity, respiratory function, ROS production, and cellular senescence.

Regarding other dynamic mitochondrial features, there is growing interest in mitochondrial quality control events in the aging of skeletal muscle.

Mitochondrial quality control involves survey, protection, and rescue strategies to limit mitochondrial damage and ensure mitochondrial integrity and function. Mitochondrial quality control involves three main steps [104]: (i) the first step that occurs at the molecular levels for the degradation of misfolded or damaged mitochondria is supported by the proteolytic system. Chaperones and ATP-dependent proteases in the matrix and inner membrane of mitochondria degrade or stabilize misfolded proteins and promote their proteolysis.

Moreover, the ubiquitin-proteasome system contributes to the quality control of mitochondria; (ii) mitochondrial fusion and fission provide a second level of quality control against mitochondrial damage. Indeed, damaged mitochondria can be repaired by fusion with healthy mitochondria, which allows the contents of healthy and dysfunctional mitochondria to be mixed. Fission, by contrast, isolates mitochondria that are irreversibly damaged or not compatible for fusion leading to their elimination by autophagy; (iii) damaged mitochondria can finally be eliminated by autophagy. In particular, mitophagy selectively removes damaged mitochondria. It requires a specific labeling of damaged mitochondria and their recruitment into isolation membranes. Thus, NIP3-like protein X (NIX; also known as BNIP3L) outer mitochondrial membrane protein binds to LC3 on the isolated membranes, which mediate the capture of damaged mitochondria in autophagosomes. Once mitochondria are damaged by losing their membrane potential, PTEN-induced putative kinase protein 1 (PINK1) recruits the E3 ubiquitin ligase parkin from the cytosol to the damaged mitochondria and causes the mitochondria to become engulfed by isolation membranes that then fuse with lysosomes. If the level of damage exceeds the capacity of all three quality control pathways, damaged mitochondria can rupture, leading to the release of proapoptotic factors and cell death.

Evidence suggests that mitophagy selectively removes malfunctioning mitochondria that are depolarized or that are producing an excessive amount of ROS. In addition, suppression of autophagy results in increased ROS production, reduced oxygen consumption, and a higher level of mtDNA mutation rates. With age, autophagy declines [105], and it has been negatively correlated with oxidative damage and apoptosis, suggesting that inhibition of autophagy may contribute to muscular atrophy and dysfunction. This has been supported by studies on muscle-specific Atg7 knockout mice, which accumulate abnormal mitochondria and have lower resting oxygen consumption and increased oxidative stress and apoptosis. These conditions positively correlated with an atrophic, weak, and degenerative phenotype [106]. Moreover, recent studies indicate that enhanced autophagy may increase lifespan [107]. Mitochondrial quality control and degradation are also controlled by the ubiquitin-proteasome system, which removes damaged proteins and short-lived proteins. Evidence from studies in mammals has suggested that ubiquitin-proteasome activity also declines with age in skeletal muscle and may be one of the causes of muscular atrophy [108]. It has recently been shown that the AMPK agonist AICAR exhibits a strong and cancer-specific growth effect, which depends on the bioenergetic signature of the cells and involves upregulation of oxidative phosphorylation. In fact, sensitivity to the pharmacological activation of AMPK is higher when cells display a high proliferation rate.

Another mechanism for eliminating damaged mitochondria is* mitoptosis*. It has been shown that in energetic stress conditions and mitochondrial network fragmentation, damaged mitochondria cluster in the perinuclear region and become incorporated into a single-membraned* mitoptotic* body and finally undergo extrusion via exocytosis or blebbing [109]. This observation is in agreement with the characterization of microvesicles carrying mtDNA released by C2C12 recently described by Guescini et al. [110].

**Figure 1:**
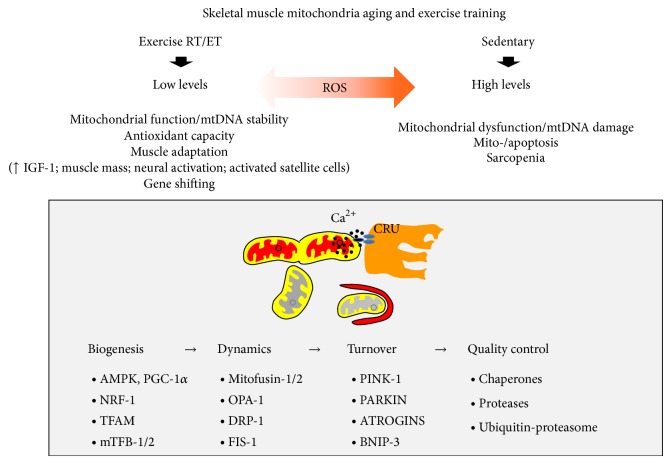
Effect of physical exercise and major signalling pathways activated on mitochondrial “quality” in aging skeletal muscle. Mitochondria represent the privileged site of ROS production. ROS may either act as signalling molecules, inducing a prosurvival response with positive muscle adaptation, or cause damage to cell components and sarcopenia. Low levels of ROS generated by skeletal muscle contraction activate a mitochondrial response that ameliorate the “quality” of skeletal muscle mitochondria cells at different molecular levels: (i)* biogenesis* through the action of the key regulators PGC-1*α*, NRF-1/2, T-FAM, and mTFB-1/2; (ii)* dynamics* by the mitochondrial remodeling GTPase proteins such as mitofusin-1/2 and OPA-1 for fusion and DRP-1 and FIS-1 for fission; (iii)* turnover* of damaged mitochondria by mitophagy through PINK-1, PARKIN, ATROGINS, and BNIP-3; and (iv) quality control by degradation of misfolded proteins or again portion of damaged mitochondria by the proteolytic system with chaperones and proteases. Slight ROS accumulation also promotes the phosphorylation state of many proteins involved in the muscle signalling responses. Moreover, low levels of ROS induced by RT play an important role in inducing upregulation of growth factors such as IGF-I. The expression of this muscle hormone has beneficial effects in muscle protein balance, muscle adaptation, and increasing muscle mass; neural activation; and number of activated satellite cells and contributes to the development of an oxidant-resistant phenotype, therefore preventing oxidative damage and chronic diseases. Moreover, the incorporation of satellite cell-derived mitochondria explains the increase in wild-type mtDNA known as “gene-shifting.” Thus, low levels of ROS elicit positive effects on muscle physiological responses. Moreover, antioxidant enzymes will function as back regulators of intracellular low ROS levels. By contrast, high levels of ROS cause functional oxidative damages of proteins, lipids, nucleic acids, and cell components and promote signalling cascades for* mitoptosis* or apoptosis. For these reasons high levels of ROS act as worsening factors in muscle atrophy, sarcopenia, and aging-related muscle diseases. Uptake of calcium by mitochondria, together with ROS, control mitochondrial quality responses in skeletal muscle cells and it is tightly regulated by sarcomeric localization and muscle chronic contraction. It occurs at calcium release unit (CRU) mitochondrion contacts where microdomains of high calcium concentration are present. RT: resistance training; CRUs: calcium release units; mtDNA: mitochondrial DNA; ET: endurance training; ROS: reactive oxygen species.

Maintaining well-functioning skeletal muscle mitochondrial dynamics in terms of content, function, and turnover is important for maintaining good health throughout our lives. Exercise stimulates key stress signals that regulate the skeletal muscle quality of mitochondria during aging (see Figure 1 for a summary). Perturbations in mitochondrial content and function can directly or indirectly impact skeletal muscle function and, consequently, the health of our whole body and overall well-being.

## 6. Exercise and Physical Activity for Older Adults

It has become increasingly clear that most of the declines in skeletal muscle function attributed to chronological age are instead a result of physical inactivity. Most physical activity guidelines for apparently healthy adults recommend performing about 30 minutes of moderate intensity aerobic exercise at least 5 times per week [10]. It has also emerged that, on a given day, these 30 minutes may be accumulated in several short bouts (of at least 10 min each) distributed throughout the day rather than in one continuous bout, without reducing training efficacy. This is a noteworthy consideration because exercising for short periods may be more pleasant and may better address common barriers to physical activity such as a lack of time or facilities, high costs, poor weather conditions, and embarrassment; hence it may improve long-term adherence to this lifestyle change. Although the minimum dose necessary to improve health status remains unclear, recently a significant improvement in cardiorespiratory fitness was reported by Mair et al. after only 4 weeks [111]. In the study, previously sedentary middle-aged adults performed 30 min per week of the bench step exercise accumulated in short bouts throughout the day. This suggests that a much shorter time than is currently recommended may be sufficient to initiate improvements in fitness.

Although ET exercise is the more common method for improving cardiovascular fitness, RT is currently recommended for the elderly, in whom loss of muscle mass and weakness are major problems. Even two training sessions a week of RT are sufficient to induce an increase in muscle strength and power [69] as a result of both an increase in muscle mass and/or in the level of neural activation.

With a similar training stimulus, the hypertrophic response seems to be blunted in older adults compared to their younger counterparts, and this phenomenon appears to be more evident in female subjects. In this regard, the role of diet, especially in terms of protein intake, remains unclear in determining muscle adaptations. In fact, it is well known that RT in elderly populations increases both mixed-muscle and MHC-specific protein synthesis to the same extent that it does in young subjects [112]. Furthermore, an increase in muscle fractional synthetic rate has been observed following RT even in frail elderly persons (>70 years) [112, 113]. RT in older adults significantly increases type II fibers CSA [54, 114, 115] as well as the proportion of type IIa fiber distribution [116].

Increasing muscle protein synthesis and muscle fiber hypertrophy corresponds to an increase in force-generating capacity and an improvement in ADL performance, leading to a significant improvement in quality of life.

RT in the elderly has also been related to decreased morbidity and even mortality [69]. The improvement in muscular strength and power induced by RT and the central and peripheral adaptations that improve cardiovascular fitness and increase energy production via oxidative metabolism induced by endurance training may be combined (i.e., concurrent training) in an efficient exercise program for the elderly that enhances both neuromuscular and cardiorespiratory functions while preserving functional ability [117]. Several studies have examined concurrent training effects on young subjects, while only few authors have investigated concurrent training adaptations in the elderly [117]. Hence, for a correct exercise prescription for the elderly it seems appropriate to identify in both exercise types the most effective combination of training variables (i.e., weekly frequency, intensity, duration, volume, exercise-order, etc.), which stimulate neuromuscular and cardiovascular adaptations to the greatest extent.

## 7. Exercise Training and Mitochondria “Quality” in the Elderly

Mitochondria play a central role in cellular metabolism and form, by fusion and fission, a dynamic reticular network within mammalian skeletal muscle [118–120]. This network allows them to share mtDNA and also to degrade and remove damaged components, through a process called mitophagy. Thus, mitochondrial biogenesis and mitophagy constantly control mitochondrial content in terms of quantity and quality [121]. Aging affects fission and fusion, mtDNA integrity, respiratory function, ROS production, cellular senescence, and also, to some extent, mitophagy. Aging also induces an increase in apoptotic cells, mostly in type II fibers [122], which contribute to sarcopenia [123].

It is well known that older adults may benefit from exercise-induced adaptations in mitochondrial biogenesis and cellular antioxidant defense [124] and that these adaptations are quickly reversed following a reduction or the cessation of physical activity.

Another important aspect to evaluate in order to understand the “trainability” of mitochondrial health and content is genetics, since there is great variability in training-induced changes in aerobic characteristics, due to nuclear genetic variants of genes [120] encoding for mt proteins and to mtDNA haplogroups [125]. The Health, Risk Factors, Training and Genetics (HERITAGE) Family Study suggests that the heritability of changes in maximal VO_2_ with exercise training is ~47% in sedentary subjects [126] while older studies in twins reported a heritability of 93.4% [127], underlining the influence of the genome on exercise-induced results.

Furthermore, studies analyzing changes in mitochondrial content and quality should also take into account fiber type and the specific mitochondrial population (i.e., SS or IMM).

As mitochondria are highly sensitivity to contractile-initiated signals, physical activity, and exercise promote biogenesis and function in these organelles, helping to maintain cellular and whole body health. In any case, many factors should be taken into consideration in determining the best exercise strategy (in terms of intensity, volume, and dosage) to improve mitochondrial function. Several studies have reported an increase in skeletal muscle mitochondrial biogenesis following ET and it appears that training volume, rather than training intensity, may be an important determinant of exercise-induced improvements in mitochondrial content [128].

RT training has been shown to counteract the deleterious effect of sarcopenia [71] and enhance mitochondrial function in aging muscle [129], but it is unclear whether the cellular adaptations to RT directly counteract the process of mitochondrial aging, interfering with key aspects of the free-radical induced aging process, or simply mask it. Since older adults rarely perform extremely high-intensity and low-repetition protocols, it is possible that mitochondrial biogenesis simply occurs in response to the volume of exercise. In any case, this issue warrants further exploration.

Wilkinson et al. [130] showed that, in young adults, a single bout of RT causes both myofibrillar and mitochondrial protein synthesis before training, while, after training, it causes only myofibrillar synthesis. Tang et al. [131] also reported an increase in mitochondrial enzyme activity following RT in young adults. In older adults, Parise et al. [114, 132] showed that RT results in high levels of antioxidant enzymes and lower oxidative stress, while Tarnopolsky [129] investigating the effects of 14 weeks of RT on oxidant status and ETC did not observe changes in the activity of complexes I+III and II+III, but they observed an upregulation of complex IV. Complex IV is the terminal electron acceptor in the ETC, and an increase in its enzymatic capacity may reduce electron leakage, leading to lower ROS production [133]. It should also be noted that complex IV possesses more proteins encoded by mtDNA than other complexes and thus is more affected by mtDNA mutations. In the skeletal muscle of older adults, as well as of some patients with mitochondrial diseases, there is heteroplasmy, which means the presence of mtDNA copies belonging to wild-type or mutant mtDNA populations. The degree of mutant mtDNA heteroplasmy found in mature skeletal muscle fibers is higher than that which is found in peripheral blood mononuclear cells, fibroblasts, and satellite cells.

Although systemic mitochondrial dysfunction plays important roles in age-related muscle wasting by preferentially affecting the quiescent myosatellite cell pool [134], satellite cells are well protected against oxidative stress. Their metabolic status is important in limiting the production of ROS [135] since they have fewer mitochondria and a glycolitic metabolism, compared to oxidative activated cells. Furthermore, quiescent satellite cells overexpress genes specifically involved in controlling the cytotoxicity of ROS and they repair DNA mutation/s much more efficiently than differentiated myoblasts. Although the repair efficiency varies slightly in different experimental conditions, satellite cells systematically repair DNA damage more efficiently than their progeny under similar conditions [136].

A process referred to as “gene shifting” can contribute to explain the benefits of RT training in improving skeletal muscle mitochondrial health. Taivassalo and colleagues [137] examined the mitochondrial genotype in mature myofibers of patients with mitochondrial disease, following either an eccentric or concentric RT intervention. They found a significant increase in the amount of wild-type mtDNA, together with a dramatic decrease in the proportion of COX-negative muscle fibers. RT causes muscle overload or injury, resulting in myofiber hypertrophy or repair processes depending on activated satellite cells that can fuse to form regenerating myofibers [138]. The incorporation of satellite cell-derived mitochondria explains the increase in wild-type mtDNA known as “gene-shifting.” More recently, Murphy and colleagues [139] showed that, in adults with large-scale mtDNA deletions, 12 weeks of RT induced an increase in muscle strength, myofiber damage and regeneration, NCAM-positive and COX-positive satellite cells, and oxidative capacity, supporting the RTe-induced mitochondrial gene-shifting hypothesis. Recently, Spendiff et al. [140], in a study on mitochondrial DNA deletion demonstrated that in patients with mitochondrial myopathies, satellite cells presented single, large-scale mtDNA deletion/s at levels comparable with those observed in muscle. According to them, whether mtDNA mutation/s occur in satellite cells, they are subsequently lost during satellite cell activation and myoblast proliferation. Thus, the gene shifting strategy induced by resistance exercise, although with a different mechanism from that originally proposed, is likely to be a suitable intervention in patients with myopathies.

This evidence supports the hypothesis that also in older adults, following RT, there is not only a mitophagic process able to remove dysfunctional mitochondria, but also a potential mtDNA-shifting. Further studies are needed to quantify the extent of this phenomenon in counteracting aging through RT and to identify the intensity of strength exercise required to activate satellite cells. This is particularly important to improve DNA-shifting and oxidative capacity, since it is known that training intensity is an important determinant in mitochondrial function and has less of an effect on mitochondrial content, which is more strongly related to training volume.

Due to the peculiar properties of ET and RT in promoting mitochondrial quantity and functioning quality, and their differing effects on biogenesis and genotype, different physical activity modalities should be combined to maximize the antiaging effects of exercise.

Thus, a preliminary period of ST which induces satellite cell activation and transfer of normal mitochondrial genes to existing muscle (decreasing the amount of COX deficient fibers and increasing the levels of wild-type compared to mutant), followed by ET induced mitochondrial biogenesis, which expands newly incorporated wild-type genomes, could be the most effective approach as suggested by [139].

According to the current paradigm, concurrent training, that is, endurance and resistance exercise combined in the same training session, results in a blunted response, due to interference between the different types of exercise. It is known that muscle growth is mediated by mTORC1, while mitochondrial biogenesis is driven by PGC-1*α*, and these two pathways are linked to one another. However, in addition to the studies supporting the interference theory, other recent studies have yielded conflicting results. Wang et al. [141] reported that endurance exercise followed by resistance exercise improves PGC-1*α* and activates mTORC1 also independently form IGF-I upregulation, its receptors. Apró et al. [142], who examined whether endurance exercise following heavy resistance exercise would repress molecular signaling through the mTORC1 pathway when compared with resistance exercise alone, did not find any interference with the growth-related signaling through the mTORC1 pathway in human skeletal muscle. In addition, both modes of exercise induced similar responses at the transcriptional level with the exception of PGC-1*α*, whose gene expression was superior following concurrent exercise. Furthermore, MacNeil et al. [143] reported that the order of exercise modes within concurrent training (endurance following resistance exercise or* vice versa*) does not affect training-induced changes in gene expression, protein content or measures of strength and aerobic capacity. Although it appears that concurrent training, regardless of the exercise mode order, is probably the most effective strategy for improving mitochondrial health and biogenesis, further studies are needed to identify the most useful exercise strategy in terms of intensity, volume, and timetable, also taking into account the overall physical condition of the subject. In this regard, the introduction of the minimally invasive technique called “fine-needle aspiration” has proved to be very useful in the study of mitochondrial quality and quantity modulation in response to exercise and may help in developing personalized exercise training programs particularly in elderly [144–146].

In addition, in subjects with an altered mitochondrial condition, extended muscle wasting and dysfunction, or nutraceutical interventions, not taken into consideration in this review, should be evaluated, alone or together with exercise. Creatine, for example, which has been shown to enhance satellite cell activation during resistance exercise training [147], could promote gene shifting. Moreover, encouraging results described in a recent meta-analysis by [148] support a role for Creatine (Cr) supplementation during RT in healthy elderly subjects. The RT with Cr supplementation enhanced muscle mass gain, strength, and functional performance, more than RT alone.

## 8. Concluding Remarks

In this review we describe the pleiotropic effect of physical activity on multiple targets that have a role in preventing the decline of mitochondrial “quality,” which is implicated in the aging process of skeletal muscle. Recent evidences consistently show that the “quality” of skeletal muscle mitochondria declines during aging. Indeed, in this condition we can observe (i) mitochondrial DNA mutations; (ii) specific epigenetic drift; (iii) decreased expression of mitochondrial proteins; (iv) reduced enzyme activity of cellular respiration; (v) reduced total mitochondrial content; (vi) increased morphological changes; (vii) a decrease in mitochondrial turnover. All of these factors probably contribute to age-associated sarcopenia, and a growing body of evidence suggests that most of these skeletal muscle age-related changes can be prevented and or attenuated by physical activity.

The current ACSM recommendations assume that older adults can safely participate in regular exercise programs (aerobic and strength). Any given exercise program for the elderly will depend on existing comorbidities and on the baseline level of physical activity. Elderly individuals can play an active role in designing their own exercise programs, which should aim to improve aerobic capacity, strength, and balance, all vital to healthy aging.

In short, physical activity should be prescribed for older adults. It not only improves physical function, helping the elderly to maintain independence, but also enhances overall health and increases longevity.
